# Electrophysiology and Structural Connectivity of the Posterior Hypothalamic Region: Much to Learn From a Rare Indication of Deep Brain Stimulation

**DOI:** 10.3389/fnhum.2020.00164

**Published:** 2020-05-15

**Authors:** Bina Kakusa, Sabir Saluja, David Y. A. Dadey, Daniel A. N. Barbosa, Sandra Gattas, Kai J. Miller, Robert P. Cowan, Zepure Kouyoumdjian, Nader Pouratian, Casey H. Halpern

**Affiliations:** ^1^Department of Neurosurgery, Stanford University School of Medicine, Stanford, CA, United States; ^2^Department of Neurosurgery, Mayo Clinic, Rochester, MN, United States; ^3^Department of Neurology and Neurosciences, Stanford University School of Medicine, Stanford, CA, United States; ^4^Department of Neurology, South Valley Neurology, Morgan Hill, CA, United States; ^5^Department of Neurosurgery, School of Medicine, University of California, Los Angeles, Los Angeles, CA, United States

**Keywords:** cluster headache, deep brain stimulation, posterior hypothalamus, local field potential, diffusion tractography

## Abstract

Cluster headache (CH) is among the most common and debilitating autonomic cephalalgias. We characterize clinical outcomes of deep brain stimulation (DBS) to the posterior hypothalamic region through a novel analysis of the electrophysiological topography and tractography-based structural connectivity. The left posterior hypothalamus was targeted ipsilateral to the refractory CH symptoms. Intraoperatively, field potentials were captured in 1 mm depth increments. Whole-brain probabilistic tractography was conducted to assess the structural connectivity of the estimated volume of activated tissue (VAT) associated with therapeutic response. Stimulation of the posterior hypothalamic region led to the resolution of CH symptoms, and this benefit has persisted for 1.5-years post-surgically. Active contacts were within the posterior hypothalamus and dorsoposterior border of the ventral anterior thalamus (VAp). Delta- (3 Hz) and alpha-band (8 Hz) powers increased and peaked with proximity to the posterior hypothalamus. In the posterior hypothalamus, the delta-band phase was coupled to beta-band amplitude, the latter of which has been shown to increase during CH attacks. Finally, we identified that the VAT encompassing these regions had a high proportion of streamlines of pain processing regions, including the insula, anterior cingulate gyrus, inferior parietal lobe, precentral gyrus, and the brainstem. Our unique case study of posterior hypothalamic region DBS supports durable efficacy and provides a platform using electrophysiological topography and structural connectivity, to improve mechanistic understanding of CH and this promising therapy.

## Background

Cluster headache (CH) is a severe trigeminal autonomic cephalalgia and often considered among the most difficult types of headaches to manage. Varying approaches have been taken in attempts to treat CH, however, poor understanding of the underlying mechanism has led to limited improvement in management (Leone et al., 2001; Vyas et al., 2019). Approximately 10% of CH patients fail medical therapy and ultimately seek surgical intervention, such as deep brain stimulation (DBS; Lovely et al., 1998). Several neuroimaging studies including tractography, positron-emission tomography, and voxel-based morphometry have linked abnormalities in hypothalamic activity to pain processing centers likely involved in CH pathogenesis (May et al., 2000; Qiu et al., 2013). DBS targeting the posterior hypothalamic region has had some success in mitigating CH although with some variability in targeting strategies across groups (Leone et al., 2001; Broggi et al., 2007; Bartsch et al., 2008; Franzini et al., 2010; Leone et al., 2010; Clelland et al., 2014). We examine a rare case of posterior hypothalamus DBS for CH to better characterize the electrophysiological properties of the posterior hypothalamus and surrounding structures. Further, we use probabilistic tractography to estimate the structural connectivity of the targeted region.

## Materials and Methods

### Clinical Data

This investigation is built upon the case of a 67-year-old, right-handed male with a long-standing history of medically refractory chronic CH in the setting of comorbid medically refractory essential tremor. The patient was primarily referred for essential tremor treatment with unilateral DBS of the ventral intermediate nucleus of the thalamus, however, before surgery, his CH was characterized by 10–120-min episodes of stabbing left-sided retro-orbital pain, ranging 8–10/10 in intensity, with 4–8 episodes per day. He failed all attempts at conservative therapy, including verapamil, prednisone, topiramate, gabapentin, indomethacin, fentanyl patches, high-flow oxygen therapy, and a sphenopalatine ganglion nerve block. An opioid dependence related to CH management was treated with buprenorphine and naloxone therapy. The patient thus consented to off-label posterior hypothalamic region DBS implantation, and this case study was approved by our institution’s internal review board (IRB#33146). A robot-assisted frameless implantation was performed as described previously (Ho et al., 2019). The left posterior hypothalamus was first targeted indirectly (*x*, *y*, *z* = 2, −5, −3 mm from the mid-commissural point) as described by others (Franzini et al., 2003; Sani et al., 2009), and confirmed by intra- and post-operative thin-cut CT imaging merged to pre-operative MRI. Microelectrode recordings were performed at 1 mm intervals on approach to the target, per our standard clinical protocol. Upon reaching the target depth, the microelectrode was removed and replaced with a Medtronic DBS lead used solely for therapeutic stimulation (Model 3387, Medtronic Inc., Dublin, Ireland).

### Electrophysiology Acquisition and Analysis

The methods of our signal acquisition and analysis pipeline have been previously described (Wu et al., 2018; Miller et al., 2019). MERs were captured at 50 kHz using Guideline 3000 MER system (Axon Instruments Inc., Foster City, CA, USA; gain, 10,000; band-pass filtered from 1 Hz to 10 kHz). In brief, 60-s recordings were captured at each depth and segmented into 5-s epochs. To extract units, the raw voltage was band-pass filtered from 3 kHz to 9 kHz and for each sample deflection above the threshold, 7 ms window from 2 ms before maximal deflection was obtained. Windowed data were decomposed using principal component analysis. Field potentials were extracted from the MER by band-pass filtering the 1 kHz downsampled data from 1 to 300 Hz. Normalized power spectral density estimates were calculated using MATLAB’s “pwelch” (50% overlap, 1–50 Hz, 2 s Hanning window) function (MATLAB 2017b, The MathWorks Inc., Natick, MA, USA). Oscillations were visually apparent and revealed by peaks in the PSD at 2 Hz (delta), 5 Hz (theta), 8 Hz (alpha), 17 Hz (beta), and 32 Hz (gamma). For each oscillation, a complex analytic signal was constructed using a band-pass (delta 1–3 Hz, theta 4–7 Hz, alpha 8–12 Hz, beta 13–25 Hz, gamma 28–50 Hz) and then applying the Hilbert transform. The rhythm phase coupling to locally measured activity was estimated by calculating the log-broadband amplitude as a function of the rhythm phase in small phase intervals (Miller et al., 2012). Finally, cross-frequency phase-amplitude coupling (PAC) was measured using the Brainstorm (v3) toolbox in MATLAB. This implementation is based on the mean-vector length modulation index as previously described for calculating a “direct PAC” measure, where a value of 0 indicates a lack of phase-amplitude modulation (Canolty et al., 2006; Özkurt and Schnitzler, 2011; Tadel et al., 2011). All implementation details are freely and readily documented and can be verified in Brainstorm’s open-source software code. For statistical analysis, a one-way analysis of variance was calculated on measured PSD between depth groups for each frequency band. Similarly, a one-way analysis of variance was calculated on measured direct PAC between depth groups for each phase-amplitude pair. Significance was determined at an alpha cutoff of 0.05 on *p*-values adjusted using the false discovery rate (FDR) method. All *p*-values reported are FDR-adjusted.

### Imaging Acquisition

Preoperatively, a T1-weighted structural 3T MRI was obtained throughout the entire cranial volume. Diffusion-weighted images (2 mm isotropic, TR/TE = 8,000/103.7 ms, 30 directions uniformly distributed on the sphere, *b* = 1,000 s/mm^2^, 300 s) were acquired from the patient. Thin-cut CT images were obtained intra (i.e., O-arm, Medtronic) and post-operatively to confirm lead placement location.

### Reconstruction of Electrodes and Volume of Activated Tissue

Lead-DBS (v2) was used to localize and visualize a 3D model of the electrodes in the patient after linear registration of the CT into the patient’s T1-weighted MRI scan (Horn et al., 2019). The co-registrations were checked by manual confirmation. The electrode trajectory was automatically reconstructed in 3D space using a search for artifacts caused by electrode leads by manually selecting the starting point of the artifact for each hemisphere. After the automatic pre-reconstruction, manual reconstruction ensued to maximize precision. The volume of activated tissue (VAT) for the bipolar stimulation modality was calculated using a finite element method based on the DBS programming parameters and the conductivity characteristics of the neural tissue activated, inferred from the contrast of the patient’s T1-weighted MRI (Horn et al., 2017).

### Diffusion Tractography

Preprocessing was performed on the diffusion-weighted images to prepare the images for tractography using Oxford FMRIB’s FSL suite. The “top-up” tool was used to estimate and correct non-zero off-resonance fields caused by the susceptibility distribution of the subject’s head. The “eddy” tool was used to correct for the eddy current caused by the rapid switching of the diffusion gradient. ROIs were generated using Freesurfer automatic segmentation to the Freesurfer subcortical atlas and the Desikan-Killiany cortical atlas (Ashburner and Friston, 2005). FSL’s Bayesian Estimation of Diffusion Parameters Obtained using Sampling Techniques (BEDPOSTX) and ProbtrackX was used to run probabilistic tractography, sampling the fiber orientation distribution in each voxel 5,000 times, accounting for crossing fibers. The seed regions for tractography were the VAT masks derived from the bipolar electrode programming parameters, with contacts 1 and 3 activated. Tractography resulted in an m × n connectivity matrix, where m refers to each voxel within the VAT, and n refers to the number of streamlines to the ROI. The voxels that did not have any streamlines to any ROI were removed. The number of streamlines from each VAT voxel was normalized by dividing the total number of streamlines between the voxels and all ROIs so that the normalized total number of streamlines is equal to 1. The proportion of streamlines from each seed voxel to each ROI were analyzed to determine significant projections. For visualization purposes, streamlines were generated using the same seeding parameters in MRtrix, and visualized in 3D using DSI Studio (Smith et al., 2013; Yeh et al., 2013).

## Results

### Post-surgical Course

**Figure 1:**
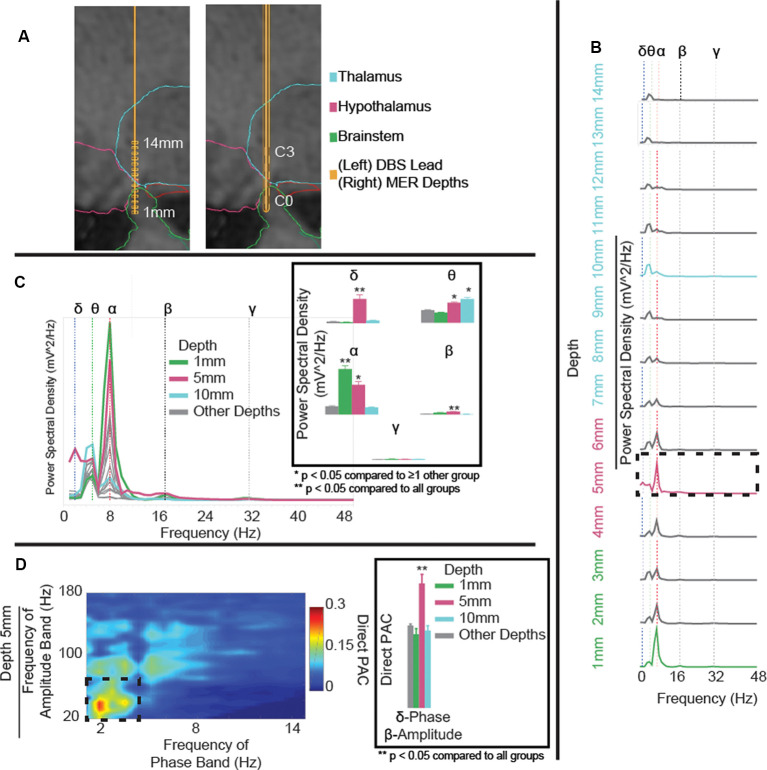
**(A)** Microelectrode recording (MER) depths (left) and deep brain stimulation (DBS) lead position (right) in T1 sagittal slice with overlaid segmented regions. Reconstruction shows DBS contacts C0 in the brainstem, C1 in the posterior hypothalamus, and C2 and C3 in the ventral anterior thalamus (VAp). **(B)** Power spectral density (PSD) estimates for field potentials collected at each depth to target, coded to the approximate region of interest. Variations in power observed in delta- (blue-dotted line), theta- (green-dotted line), alpha- (red-dotted line), beta- (black-dotted line), and gamma-frequency bands (gray-dotted line). Delta- and alpha-band powers increase with proximity to the posterior hypothalamic region. **(C)** Aggregated PSD estimates for field potentials collected at each depth to target, coded to the approximate region of interest. Panel **(C)** highlighting significant (*p* < 0.001) delta power in the posterior hypothalamic region (5 mm), increased theta at 5 and 10 mm, and increased alpha at 1 and 5 mm from the target. **(D)** Cross-frequency phase-amplitude coupling (PAC), using the direct PAC measure. Panel **(D)** highlighting significant (*p* < 0.001) delta-band phase coupling to beta-band amplitude in the posterior hypothalamic region (5 mm), not present at any other depth (Supplementary Figure S1). **p < 0.05 when compared to all other depth groups, *p < 0.05 when compared to ≥1 other group.

Two weeks postoperatively, the patient was seen for initial programming. Contacts were found to be in the midbrain (contact 0, C0), posterior hypothalamus (C1), and ventral anterior thalamus (VAp; C2 and C3; Figure 1A). Monopolar testing of the lead contacts revealed decreased headache on activation of contact 2 (C2). Follow-up testing found that bipolar stimulation of contacts 1–2 (0.5V, C1− C3+, 60 ms, 165 Hz) resulted in the most robust response with no discernable headache symptoms. At 18 months post-surgically, active contacts were unchanged but stimulation parameters were slightly modified due to modest recurrence of symptoms (1.2V, C1− C3+, 60 ms, 100 Hz). At this time, the patient endorsed a lack of headache symptoms and no adverse side effects (Figure 1B). The patient also experienced expectedly unrelated relief of his essential tremor with activation of his thalamic lead (data not shown).

### Intraoperative Electrophysiology and Tractography

MER started 14 mm from the hypothalamic region target (*x*, *y*, *z* = 2, −5, −3 mm from the mid-commissural point) along the pre-planned trajectory and they were collected at 1 mm interval advancements (Figure 1A). The microelectrode was then removed and replaced with a DBS lead along the same trajectory. As a single trajectory was used for both MER and DBS lead placement, reconstruction of MER positions was extrapolated from the measured lead depth. Overall, theta- and alpha-band power increased with proximity to the hypothalamic region (alpha-band: *F*_(1,3)_ = 103.1, *p* < 0.0001, 1 mm vs. rest: *p* < 0.0001, 5 mm vs. 10 mm/other depths: *p* < 0.0001; Figure 1B). Comparing power across depths, alpha and beta-band power showed local maxima at depths of 1 mm and 5 mm (C1) from target while theta-power maxima were observed at depths of 5 mm (C1), and 10 mm (C3) from target (beta-band: *F*_(1,3)_ = 43.6, *p* < 0.0001, 5 mm vs. rest *p* < 0.0001; theta-band: *F*_(1,3)_ = 33.4, *p* < 0.0001, 5 mm vs. 1 mm/other depths: *p* < 0.0001, 10 mm vs. 1 mm/other depths: *p* < 0.0001; Figure 1C). Delta-band power had one clear peak at 5 mm (C1) from target (delta-band: *F*_(1,3)_ = 130, *p* < 0.0001, 5 mm vs. rest: *p* < 0.0001). Finally, notable delta-band phase and beta-band amplitude coupling was observed at 5 mm (C1) but not at any other depth (delta-beta PAC: *F*_(1,3)_ = 14.5, *p* < 0.0001, 5 mm vs. rest: *p* < 0.0001; Figure 1D and Supplementary Figure S1). Also, theta-band phase and gamma-band amplitude coupling was increased at 5 mm compared to 1 mm and other depths but not 10 mm (theta-gamma PAC: *F*_(1,3)_ = 4.5, *p* = 0.004, 5 mm vs. 1 mm/other depths: *p* < 0.05).

**Figure 2:**
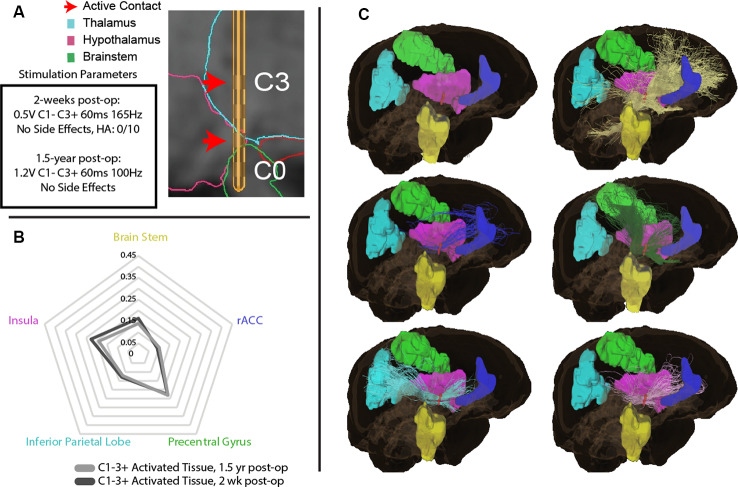
**(A)** Initial parameters at 2-weeks post-operation and 1.5-year post-surgically with DBS lead depicting active contacts in green. **(B)** Regions of interest (ROIs) with greatest tractography-based proportion of streamlines (>5% of normalized streamlines) to the volume of activated tissue (VAT) with parameters at 2-weeks post-operation, and 1.5-years post-surgery. **(C)** Visual representation of ROIs with greatest tractography-based proportion of streamlines to the brain stem (yellow), rostral anterior cingulate cortex (rACC, blue), precentral gyrus (green), inferior parietal lobe (blue), insula (pink), and brain stem (yellow).

To explore the white matter fiber tracts of the VAT, tractography was performed from the VAT calculated for initial parameters at 2 weeks (bipolar, C1−C3+), and parameters at 1.5 years (Figure 2A). The initial 2-week programming parameters of bipolar stimulation at C1− and C3+ resulted in a VAT that had prominent streamlines (>5%) to the insula, rostral anterior cingulate cortex (rACC), inferior parietal lobe, precentral gyrus, and brain stem (Figures 2B,C). The VAT resulting from parameters at 1.5 years had nearly an identical streamline profile to that at 2-weeks postoperatively.

## Discussion

This investigation sheds light on the mechanisms of a successful DBS treatment of CH. Electrophysiological mapping along the posterior hypothalamus trajectory suggests a topological correspondence between field potential activity and the position of clinically therapeutic contacts. Specifically, delta- (3 Hz) and alpha-band (8 Hz) powers increase with proximity to this region with significant coupling between delta- and beta-band (17 Hz). Further, the VAT encompassing this region has a high proportion of streamlines to regions associated with pain processing (May et al., 2000; Qiu et al., 2013).

Targeting of regions including the posterior hypothalamus and selection of active contacts largely relies on results from prior treatment trials and trial and error during intraoperative and post-operative stimulation testing. Regional electrophysiology, namely readily recordable field potentials, and VAT tractography models provide a potential bridge to optimizing both preoperative planning and intraoperative targeting and in aiding in postsurgical programming (Chen et al., 2006; Thompson et al., 2014; Tinkhauser et al., 2018; Akram et al., 2017). In the case of the subthalamic nucleus in Parkinson’s, field potential biomarkers have been reported to be predictive of contacts with greater therapeutic effect upon stimulation (Chen et al., 2006). In the case of the posterior hypothalamus in CH, electrophysiological mapping of the region has largely been limited to recordings in a small number of case series, largely focused on reporting single-unit firing rates (Bartsch et al., 2008; Cordella et al., 2007, 2010; Starr et al., 2007; Micieli et al., 2017; Sani et al., 2009).

To our knowledge, only two studies have reported on field potentials in the posterior hypothalamus. Nager et al. (2011) demonstrated event-related field potential differences to motivational (sexually-relevant and food) stimuli compared to common control items, however, they did not characterize frequency-band specific results (Nager et al., 2011). Brittain et al. (2009) demonstrated that during a CH attack, there was a significant increase in beta-band (20 Hz) field potential power, representing a possible pathologically-relevant biomarker. Here, we demonstrate field potential topography in and around the hypothalamic region with the increasing prominence of low frequency- (delta-, and alpha-) band power with proximity to this region. While recordings here were performed outside of a CH attack episode, we find prominent delta- and beta-band coupling in the hypothalamic region, highlighting the previously demonstrated pathologic association. This marker could be utilized in localizing an appropriate depth to target the region in future implantations. However, the spectral findings in this study are limited by a lack of control data to delineate their specificity as a pathologically-relevant biomarker. Further work is needed including capturing spectral changes during CH attacks and in non-CH patients to better characterize this activity and explore its utility during intraoperative targeting and post-surgical programming.

The pathological basis of CH is poorly understood but many studies have demonstrated aberrant activity in some pain processing regions. Although the results from this investigation are representative of a single case of CH, our analysis sheds light on the possible mechanisms of CH pathogenesis *via* the pain-matrix network and central autonomic matrix. Painful stimuli elicit activation upon the pain-matrix network of cortical structures, such as somatosensory, insular, and cingulate areas. Activation of this network has been demonstrated in multimodal investigations, suggesting that it functions as a salience detection system for pain (Legrain et al., 2011). Moreover, functional MRI studies have suggested two major groups of structures to be involved in CH—the pain-matrix network structures, and separately, the hypothalamus (May and Goadsby, 1999). In the case of the central autonomic network, dysfunctional autonomic regulation, with the hypothalamus as a key mediator, contributes to the pathological mechanism of CH with autonomic symptoms including lacrimation, ptosis, and conjunctival injection present during attacks (May, 2005). Our results provide further evidence of an association between these structures through prominent streamlines from the hypothalamic VAT that may modulate or be modulated by structures in the pain-matrix and central autonomic networks.

Notably in this study, the ACC, insula, and brainstem received a high proportion of streamlines to the VAT associated with clinical improvement. Within the pain-matrix network, the ACC and insula are thought to be centers for affective components of pain processing and perception and modulation of pain, respectively (Fuchs et al., 2014; Lu et al., 2016). Within the central autonomic network, the ACC, insula, and several brain stem regions are key nodes in the autonomic nervous system function (Sklerov et al., 2018). Streamlines to the precentral gyrus and the inferior parietal lobe were prominent with our parameters. These findings are consistent with previously reported structural and functional abnormalities in cluster headache patients, which have helped to shed light on their possible pathologic roles. In CH patients, the precentral gyrus was found to have decreased cortical thickness (Cosentino et al., 2015; Seifert et al., 2012). It has also been reported that CH patients possessed decreased gray matter volume in structures in the pain-matrix network, notably the precentral gyrus, insula, and inferior parietal lobe (Absinta et al., 2012). The inferior parietal lobe has been reported to have increased resting-state functional connectivity to the posterior hypothalamus in CH patients that further increases during painful episodes (Qiu et al., 2013; Chou et al., 2017). Also, repetitive transcranial magnetic stimulation of the precentral gyrus has been used to successfully deliver therapeutic benefit to CH patients (Cosentino et al., 2015; Hodaj et al., 2015). DBS modulation of pain processing pathways is further supported by recent studies showing that responders to posterior hypothalamus stimulation have VAT along the trigeminohypothalamic pathway and associations between the posterior hypothalamus with other regions for autonomic regulation and pain (May et al., 2006; Akram et al., 2017). Put together, the findings from this investigation bring a new perspective with direct neural recording, imaging, and direct modulation results that helps to elucidate the mechanisms of DBS in the context of the treatment of CH. Future studies can probe the extent that these spectral and tractographic features predominate in CH attack vs. non-attack posterior hypothalamic region activity and how these features are modulated by DBS.

## Data Availability Statement

The datasets generated for this study are available on request to the corresponding author.

## Ethics Statement

The studies involving human participants were reviewed and approved by Stanford University School of Medicine Institutional Review Board. The patients/participants provided their written informed consent to participate in this study.

## Author Contributions

CH, KM, DD, and BK contributed to the conception and design of the study. BK, SS, and DB performed the statistical analysis. All authors contributed to the drafting and revision of the manuscript, and approved the submitted version.

## Conflict of Interest

CH receives speaking honoraria and consulting fees from NeuroPace, Medtronic, and Boston Scientific. The remaining authors declare that the research was conducted in the absence of any commercial or financial relationships that could be construed as a potential conflict of interest.

## Supplementary Material

FIGURE S1 **(A)** Cross-frequency phase-amplitude coupling at each microelectrode depth recording revealing prominent delta-band phase coupling to beta-band amplitude in the posterior hypothalamic region (5 mm), not present at any other depth. **(B)** Non-zero direct PAC measure for each phase-amplitude pair by depth group highlighting significant delta-phase to beta-amplitude coupling in the hypothalamic region. ***p* < 0.05 when compared to all other depth groups, **p* < 0.05 when compared to ≥1 other group.
