# Supplementary figures and images for: Electrophysiology and Structural Connectivity of the Posterior Hypothalamic Region: Much to Learn From a Rare Indication of Deep Brain Stimulation
Source: Front Hum Neurosci. 2020 May 15;14:164. doi: 10.3389/fnhum.2020.00164 (PMC7326144; doi:10.3389/fnhum.2020.00164)

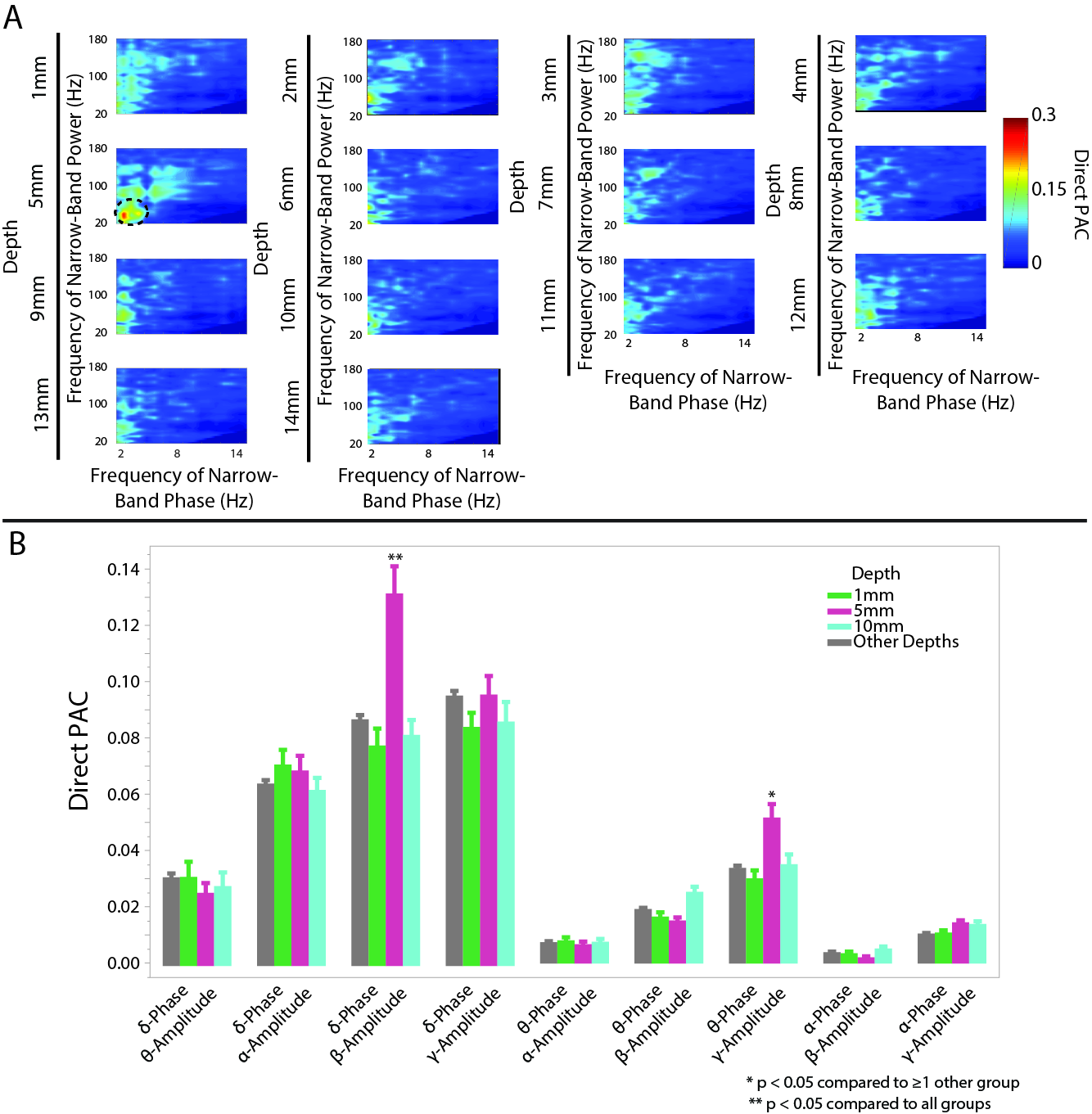

Supplement: FIGURE S1 — (A) Cross-frequency phase-amplitude coupling at each microelectrode depth recording revealing prominent delta-band phase coupling to beta-band amplitude in the posterior hypothalamic region (5 mm), not present at any other depth. (B) Non-zero direct PAC measure for each phase-amplitude pair by depth group highlighting significant delta-phase to beta-amplitude coupling in the hypothalamic region. **p < 0.05 when compared to all other depth groups, *p < 0.05 when compared to ≥1 other group. [file Image_1.tif]
